# Functional Characterization of Glycoprotein Nonmetastatic Melanoma Protein B in Scleroderma Fibrosis

**DOI:** 10.3389/fimmu.2022.814533

**Published:** 2022-02-24

**Authors:** Pamela J. Palisoc, Leah Vaikutis, Mikel Gurrea-Rubio, Ellen N. Model, Morgan M. O’mara, Sarah Ory, Sirapa Vichaikul, Dinesh Khanna, Pei-Suen Tsou, Amr H. Sawalha

**Affiliations:** ^1^ Division of Rheumatology, Department of Internal Medicine, University of Michigan, Ann Arbor, MI, United States; ^2^ Scleroderma Program, University of Michigan, Ann Arbor, MI, United States; ^3^ Division of Rheumatology, Department of Pediatrics, University of Pittsburgh School of Medicine, UPMC Children’s Hospital of Pittsburgh, Pittsburgh, PA, United States; ^4^ Division of Rheumatology and Clinical Immunology, Department of Medicine, University of Pittsburgh School of Medicine, Pittsburgh, PA, United States; ^5^ Lupus Center of Excellence, University of Pittsburgh School of Medicine, Pittsburgh, PA, United States

**Keywords:** scleroderma, fibrosis, dermal fibroblasts, GPNMB, autoimmunity

## Abstract

Glycoprotein nonmetastatic melanoma protein B (GPNMB) is involved in various cell functions such as cell adhesion, migration, proliferation, and differentiation. In this study, we set forth to determine the role of GPNMB in systemic sclerosis (SSc) fibroblasts. Dermal fibroblasts were isolated from skin biopsies from healthy subjects and patients with diffuse cutaneous (dc)SSc. GPNMB was upregulated in dcSSc fibroblasts compared to normal fibroblasts, and correlated negatively with the modified Rodnan skin score. In addition, dcSSc fibroblasts secreted higher levels of soluble (s)GPNMB (147.4 ± 50.2 pg/ml vs. 84.8 ± 14.8 pg/ml, p<0.05), partly due to increased ADAM10. sGPNMB downregulated profibrotic genes in dcSSc fibroblasts and inhibited cell proliferation and gel contraction. The anti-fibrotic effect of sGPNMB was at least in part mediated through CD44, which is regulated by histone acetylation. TGFβ downregulated GPNMB and decreased the release of its soluble form in normal fibroblasts. In dcSSc fibroblasts, GPNMB is upregulated by its own soluble form. Our data demonstrate an anti-fibrotic role of sGPNMB in SSc and established a role for the ADAM10-sGPNMB-CD44 axis in dermal fibroblasts. Upregulating GPNMB expression might provide a novel therapeutic approach in SSc.

## Introduction

Systemic sclerosis (scleroderma, SSc) is an autoimmune disease that affects the immune and vascular systems, and causes fibrosis in multiple organs. Current treatment options for this disease are limited, predominantly relying on immune suppression with the goal to slow down disease progression. Progressive organ fibrosis, specifically in the lungs, is the leading cause of death in SSc (1). Fibrosis, which is commonly caused by fibroblast activation, is a pathogenic condition characterized by myofibroblast transformation, increased collagen/extracellular matrix (ECM) production, and reduction of ECM turnover.

Glycoprotein nonmetastatic melanoma protein B (GPNMB), also known as osteoactivin or dendritic cell-associated heparan sulfate proteoglycan-integrin ligand (DC-HIL), is a highly glycosylated protein that is localized on cell membranes or stored in endosomes and lysosomes (2). It is widely expressed in many cell types and has a wide array of functions that are critical for various physiological and pathological processes. Many of these functions are also mediated by its soluble form, which is produced by cleavage of the membrane-bound GPNMB by metalloproteinases such as ADAM10 (3). In stromal cells, soluble (s)GPNMB has been shown to mediate its effect through engaging with CD44, a transmembrane glycoprotein involved in cell-matrix or cell-cell interactions (2).

GPNMB has been implicated in tissue remodeling and repair processes after tissue injury. Although it is associated with tumor progression and metastasis, its’ role appears to be protective in tissue homeostasis. It has been reported that GPNMB limits inflammation and favors protection in the bone and brain (4–6). In tissue repair, macrophage-derived sGPNMB promoted wound healing by recruiting mesenchymal stem cells (7). Interestingly, GPNMB overexpression in transgenic animals was protective against diet-induced liver fibrosis (8). Soluble GPNMB was also able to activate signaling pathways and induced matrix metalloproteases in fibroblasts (9). Considering its effect on wound healing and fibrosis, in this study we determined the function of GPNMB in SSc fibrosis in patient-derived dermal fibroblasts. We also established the regulatory network for sGPNMB in these cells.

## Materials and Methods

### Subject Enrollment

Both healthy individuals and patients with diffuse cutaneous SSc (dcSSc) (10) were recruited. This study was approved by the University of Michigan Institutional Review Board. Characteristics of the enrolled study participants are summarized in **Table 1**.

**Table 1:** SSc patients and healthy controls characteristics.

	dcSSc (n = 51)	Healthy volunteers (n = 41)
Age (years)	58 (49-67)^a^	52 (38-72)^a^
Sex	F35/M16	F27/M14
Disease duration (years)	2 (1-4)^a^	N.A.
Modified Rodnan skin score	18 (12-26)^a^	N.A.
Raynaud’s phenomenon	49	N.A.
Early disease (< 5yrs)	48	N.A.
Interstitial lung disease	33	N.A.
Pulmonary arterial hypertension	12	N.A.
Immunosuppressives	42	N.A.

a Median (Interquartile range).

N.A., Not applicable.

### Cell Culture

Dermal fibroblasts were isolated from punch biopsies obtained from healthy subjects and dcSSc patients as previously described (11–13). Cells between passages 3 and 6 were maintained in RPMI supplemented with 10% fetal bovine serum (FBS) and antibiotics.

### Enzyme‐Linked Immunosorbent Assay (ELISA)

The levels of sGPNMB in cell culture supernatants and plasma were measured using an ELISA kit from R&D Systems. Absorbance at 450 nm in each well was read using a microplate reader. Cell culture media were changed to RPMI with 1% FBS and cultured for 48 hours before they were collected for analysis.

### qRT-PCR

Extraction of total RNA was done using the Direct-zol™ RNA MiniPrep Kit (Zymo Research) followed by cDNA synthesis using the Verso cDNA Synthesis Kit (Thermo Fisher). Quantitative PCR was performed using a ViiA™ 7 Real-Time PCR System. Primers for *GPNMB*, *CD44*, *COL1A1*, *ACTA2*, *ADAM10*, and *ACTB* were used in this assay.

### Western Blots

Cells grown in 6-well plates were lysed in RIPA buffer containing protease inhibitors. The blots were probed with antibodies against collagen I (COL1, Abcam), α-smooth muscle actin (αSMA, Abcam), ADAM10 (Novus), CD44, and GPNMB (both from Cell Signaling Technology). For loading control, the blots were immunoblotted with antibodies against β-actin (Sigma) or GAPDH (Cell Signaling Technology). Band quantification was performed using ImageJ (14).

### Immunofluorescence Staining

Cells were fixed in 4% formalin and blocked. They were then probed with anti-GPNMB antibodies (R&D Systems). Alexa Fluor antibodies (Invitrogen) were subsequently used. The nuclei were stained using DAPI (Invitrogen). Fluorescence was detected using a Nikon A1 confocal microscope. Visualization and analysis of images were performed using the ND2 reader plugin in ImageJ.

### Cell Treatment and Transfection

Dermal fibroblasts from dcSSc patients were treated with 0.01-100 ng/ml of sGPNMB (R&D Systems) for 72 hours. In separate experiments, fibroblasts were treated with BET inhibitor JQ1 (1µM, Cayman Chemicals) or HDAC inhibitor Panobinostat (1µM, Selleck Chemicals) for 48 hours. To knockdown CD44 in dcSSc dermal fibroblasts, small interfering RNA (siRNA) towards human CD44 (1 µM, Accell siRNA, Dharmacon), was used. Scrambled siRNA (Dharmacon) was used as a control. The cells were transfected for 96 hours before downstream experiments were performed. To induce a myofibroblast phenotype, normal dermal fibroblasts were treated with TGFβ (10 ng/ml) for 72 hours. Inhibition of ADAM10 was achieved by incubating the cells with 10 µM of GI254023X (Cayman Chemical) for 48 hours.

### Cell Proliferation Assays

The IncuCyte^®^ Live-Cell Imaging System was used to monitor cell proliferation. Cells were seeded at 5,000 cells/well and allowed to grow overnight. After adding sGPNMB at different concentrations (0-100 ng/ml), cells were monitored by IncuCyte^®^. Cell counts were analyzed by the IncuCyte^®^ S3 Analysis software.

### Gel Contraction and Cell Migration Assays

The Cell Contraction Assay Kit (Cell Biolabs) was used to examine gel contraction by sGPNMB treatment. Gels were lifted after 24 hours and the areas of the gels were quantified using ImageJ (14). For wound healing, we performed a scratch wound assay using the IncuCyte^®^ Live-Cell Imaging System. Fibroblasts were grown to confluence and a wound gap was created using the IncuCyte^®^ Woundmaker. After washing the cells, culture media with sGPNMB (0-100 ng/ml) was added. Cell migration was monitored and analyzed by IncuCyte^®^ S3 Analysis software.

### Statistical Analysis

To determine the differences between groups, Mann–Whitney U test, Wilcoxon test, Kruskal–Wallis test, or two-way ANOVA were performed using GraphPad Prism version 8 (GraphPad Software, Inc). Pearson or Spearman correlation coefficient were used for correlation analysis. P values of less than 0.05 were considered statistically significant. Results were expressed as mean ± SD.

## Results

### GPNMB Expression in SSc

**Figure 1:**
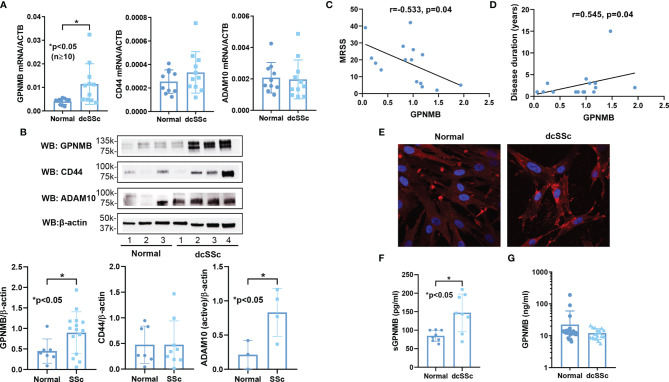
GPNMB, CD44, and ADAM10 expression in normal and dcSSc fibroblasts. **(A)** *GPNMB* mRNA levels were significantly upregulated in dcSSc fibroblasts compared to healthy controls, while *CD44* and *ADAM10* levels were similar. **(B)** At the protein level, differences in CD44 did not reach statistical significance, while GPNMB and ADAM10 were significantly increased in dcSSc fibroblasts. **(C)** GPNMB protein levels in dcSSc fibroblasts negatively correlated with the modified Rodnan skin score (MRSS). **(D)** GPNMB protein levels in dcSSc fibroblasts positively correlated with disease duration. **(E)** Immunofluorescence staining of GPNMB in both normal and dcSSc fibroblasts showed that GPNMB was widely expressed in the cytosol. In dcSSc fibroblasts GPNMB showed prominent staining at the plasma membrane. **(F)** The soluble form of GPNMB was significantly higher in culture media from dcSSc fibroblasts compared to healthy controls. **(G)** Serum levels of sGPNMB were similar between healthy controls and dcSSc patients. n=number of subjects. Results are expressed as mean +/- SD and p < 0.05 was considered significant.

We observed significant elevation of GPNMB mRNA and protein levels in dcSSc fibroblasts compared to controls (**Figures 1A, B**). In addition, we found that GPNMB protein levels were negatively correlated with the modified Rodnan skin score (MRSS, **Figure 1C**), and positively correlated with disease duration (**Figure 1D**). GPNMB levels were also negatively correlated with the MRSS at the biopsy site (r=-0.495, p=0.06). To visualize the cellular location of GPNMB in dermal fibroblasts, we performed immunofluorescence in both normal and dcSSc fibroblasts. It appears that GPNMB is localized to the cytoplasm in dermal fibroblasts, possibly within lysosomal or endosomal compartments. In addition, prominent staining of GPNMB on the plasma membrane of dcSSc fibroblasts was observed (**Figure 1E**). Since GPNMB can be cleaved by ADAM10 into a soluble form, we also measured sGPNMB in culture media. We found that dcSSc fibroblasts produced significantly higher amounts of sGPNMB than normal fibroblasts (**Figure 1F**). In plasma samples, we did not observe any differences in sGPNMB levels between healthy individuals and dcSSc patients (**Figure 1G**).

### The Anti-Fibrotic Effect of sGPNMB in dcSSc Fibroblasts

**Figure 2:**
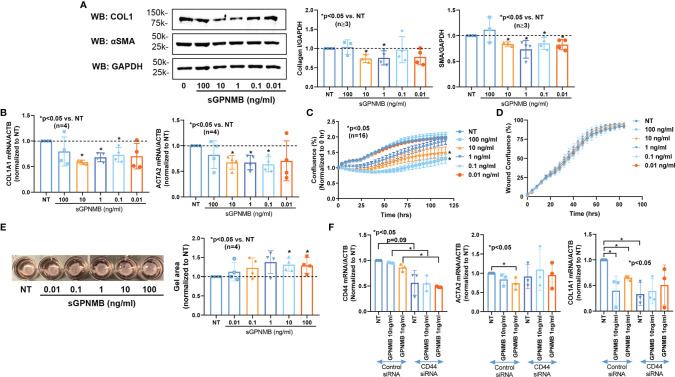
sGPNMB inhibits SSc fibrosis *in vitro*. **(A)** sGPNMB significantly reduced COL1 and αSMA expression in dcSSc fibroblasts at various doses. **(B)** sGPNMB downregulated *COL1A1* and *ACTA2* at the mRNA level in dcSSc fibroblasts. **(C)** sGPNMB significantly reduced cell proliferation of dcSSc fibroblasts at a dose-dependent manner. Cell growth was monitored by IncuCyte^®^ Live-cell imaging. **(D)** Migration of dcSSc fibroblasts was not significantly affected by sGPNMB. Cell migration was monitored by IncuCyte^®^ Live-cell imaging. Wound confluence indicates the number of cells migrated into the wound gap. **(E)** Gel contraction by dcSSc fibroblasts was inhibited by sGPNMB at various doses. **(F)** The anti-fibrotic effect of sGPNMB was absent in CD44 knocked down dcSSc fibroblasts, as shown by *COL1A1* and *ACTA2* levels. n=number of subjects. Results are expressed as mean +/- SD and p < 0.05 was considered significant.

Since dcSSc fibroblasts release significantly higher levels of sGPNMB, we examined the effect of sGPNMB on dcSSc fibroblasts. We found that sGPNMB significantly reduced fibrotic markers including COL1 (at 1 and 10 ng/ml) and αSMA (at 0.01-10 ng/ml) in dcSSc fibroblasts (**Figure 2A**). This was also shown at the mRNA level; sGPNMB, in concentrations between 0.1-10 ng/ml, significantly downregulated *COL1A1* and *ACTA2* (**Figure 2B**). In addition, sGPNMB dose-dependently inhibited proliferation in dcSSc fibroblasts (**Figure 2C**) while it had minimal effect on cell migration (**Figure 2D**). In the gel contraction assay, sGPNMB at concentrations of 10 and 100 ng/ml significantly relaxed contraction, as shown by the increase in gel surface area (**Figure 2E**). These results suggest that sGPNMB is anti-fibrotic in dcSSc dermal fibroblasts.

### sGPNMB Activates CD44 Upon Being Cleaved by ADAM10 in dcSSc Fibroblasts

It has been reported that sGPNMB is produced by cleavage of its membrane form by ADAM10, thereby acting on osteoclasts, mesenchymal stem cells, and adipocytes by engaging with a transmembrane glycoprotein CD44 (2, 3, 15). CD44 and ADAM10 were expressed in both normal and dcSSc fibroblasts (**Figures 1A, B**). There were no differences in CD44 levels between normal and dcSSc fibroblasts, while ADAM10 was significantly increased in dcSSc fibroblasts at the protein level (**Figure 1B**). As the sGPNMB-CD44 axis in dermal fibroblasts has not been previously explored, we examined whether CD44 knockdown in dcSSc fibroblasts affects the anti-fibrotic effects of sGPNMB. We found that knockdown of CD44 in these cells led to significant downregulation of *COL1A1* while it had no effect on *ACTA2* (**Figure 2F**). sGPNMB did not downregulate *COL1A1 *and* ACTA2* in CD44 knocked down cells compared to cells transfected with control siRNA. While the change of *COL1A1* at baseline made it difficult to interpret the effect of sGPNMB on collagen production, the effect of sGPNMB on *ACTA2* was clear, confirming that CD44 is indeed a cell surface receptor for sGPNMB.

### TGFβ Downregulates GPNMB and CD44, While Upregulates ADAM10 in Normal Fibroblasts

**Figure 3:**
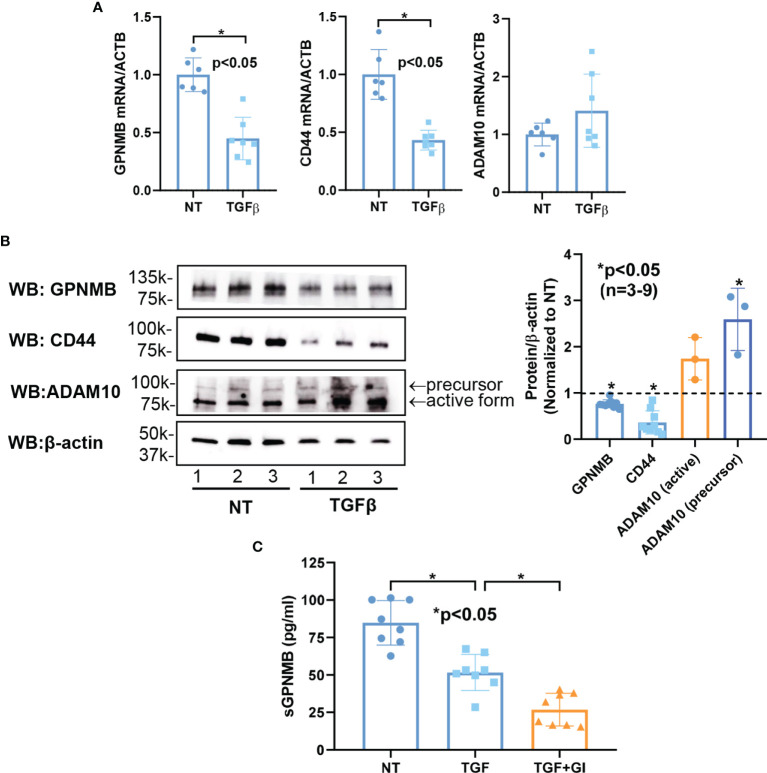
Effect of TGFβ on GPNMB, CD44, and ADAM10 in normal dermal fibroblasts. **(A)** TGFβ significantly downregulated both *GPNMB* and *CD44* in normal fibroblasts, but had minimal effect on *ADAM10* expression. **(B)** At the protein level, significantly lower levels of GPNMB and CD44 were observed after TGFβ treatment. In contrast, ADAM10, specifically the precursor form, was significantly upregulated by TGFβ. **(C)** The soluble form of GPNMB was significantly lower in culture media from TGFβ-treated fibroblasts. Inhibition of ADAM10 by GI254023X (GI) further reduced the amount of sGPNMB in culture media. n=number of subjects. Results are expressed as mean +/- SD and p < 0.05 was considered significant.

To investigate the mechanisms that regulate the expression of GPNMB, CD44, and ADAM10 in dermal fibroblasts, we stimulated normal dermal fibroblasts with TGFβ to induce a myofibroblast phenotype. As shown in **Figure 3A**, TGFβ significantly downregulated both *GPNMB* and *CD44* mRNA levels, but had no effect on *ADAM10*. Similarly, at the protein level, both GPNMB and CD44 were downregulated by TGFβ (**Figure 3B**). As for ADAM10, TGFβ appears to stimulate its expression, in particular the precursor form. We further examined whether TGFβ affects the soluble form of GPNMB. As shown in **Figure 3C**, TGFβ treatment in normal fibroblasts for 3 days significantly reduced sGPNMB release from these cells. In addition, inhibition of ADAM10 by GI254023X significantly lowered sGPNMB to a greater extent.

### sGPNMB Forms a Positive Feedback Loop in dcSSc Fibroblasts

**Figure 4:**
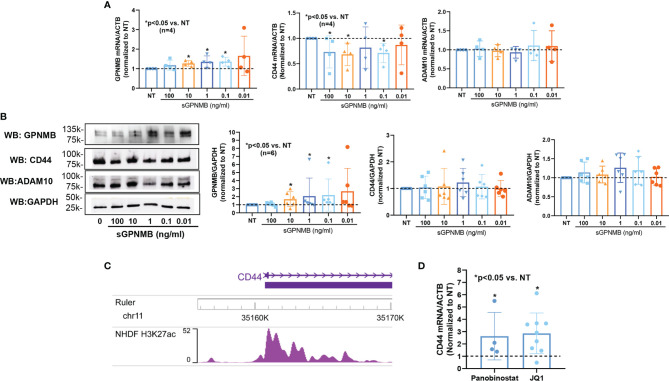
Mechanisms involved in GPNMB and CD44 expression in dcSSc fibroblasts. **(A)** sGPNMB significantly induced *GPNMB* expression in dcSSc fibroblasts and downregulated *CD44* expression. sGPNMB had minimal effect on *ADAM10* expression. **(B)** At the protein level, sGPNMB induced GPNMB at 0.1, 1, and 10 ng/ml while it had no effect on CD44 and ADAM10. **(C)** Genome browser track of the *CD44* locus along with ChIP-seq data for H3K27ac in normal human dermal fibroblasts (NHDF) generated by the ENCODE project. **(D)** CD44 levels were elevated after dcSSc fibroblasts were treated with HDAC inhibitor Panobinostat or histone reader inhibitor JQ1. n=number of subjects. Results are expressed as mean +/- SD and p < 0.05 was considered significant.

To further examine the mechanism involved in elevated GPNMB levels in dcSSc fibroblasts, we examined the effect of sGPNMB on *GPNMB*, *CD44*, and *ADAM10* in these cells. Interestingly, sGPNMB induced *GPNMB* in dcSSc fibroblasts and suppressed *CD44*. sGPNMB had minimal effect on *ADAM10* expression (**Figure 4A**). At the protein level, sGPNMB indeed induced GPNMB expression but had minimal effect on CD44 and ADAM10 (**Figure 4B**). These results suggest that sGPNMB has the ability to regulate its own expression. The elevated levels of GPNMB in dcSSc fibroblasts are potentially caused by a self-induced positive feedback loop.

### CD44 Is Regulated by Histone Acetylation in dcSSc Fibroblasts

To determine whether CD44 is regulated by epigenetic mechanisms, we extracted histone 3 lysine 27 acetylation (H3K27ac) ChIP-seq data from ENCODE generated using normal human dermal fibroblasts (NHDF). There was increased peak intensity of H3K27ac at the promoter region of *CD44* (**Figure 4C**). We then treated dcSSc fibroblasts with a pan-HDAC inhibitor (Panobinostat) or a BET histone reader inhibitor (JQ1). *CD44* expression was elevated significantly by both inhibitors, suggesting that CD44 expression is indeed regulated by histone acetylation in these cells (**Figure 4D**).

## Discussion

**Figure 5:**
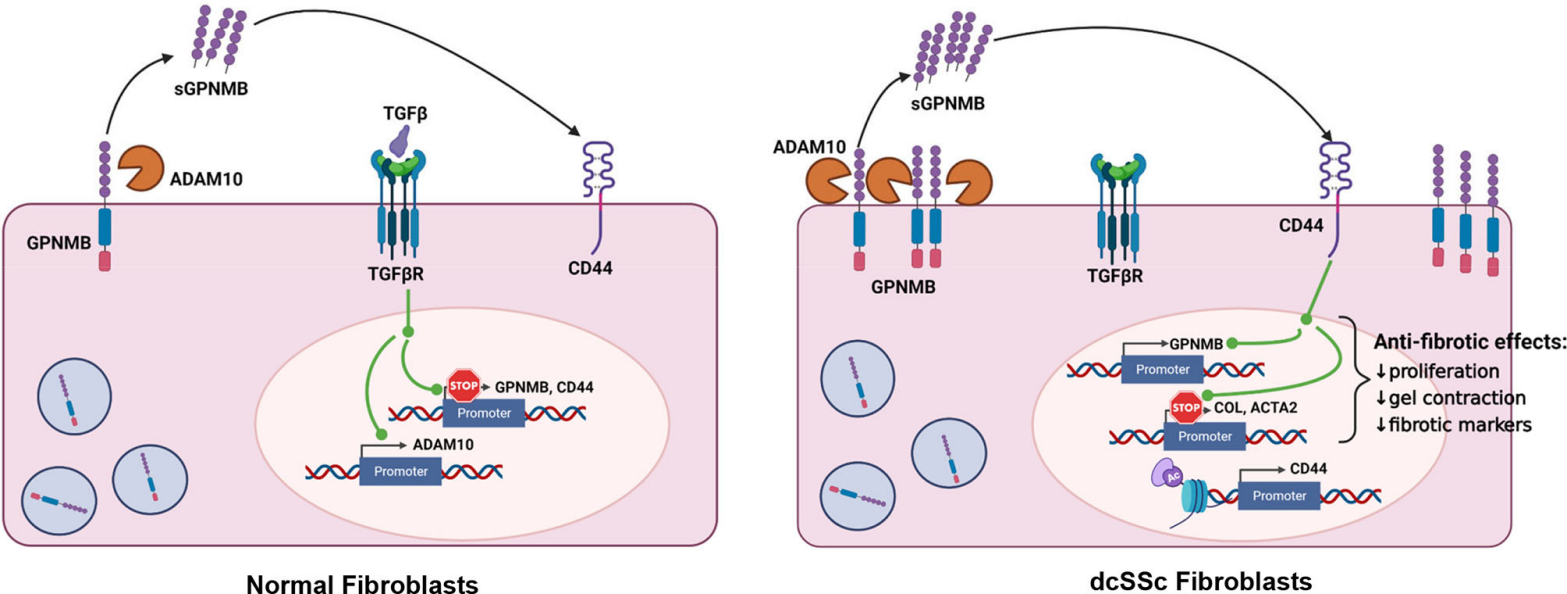
Summary of this study. In normal fibroblasts, ADAM10 cleaves off the soluble form of GPNMB. TGFβ downregulates GPNMB and CD44, and upregulated ADAM10. In dcSSc fibroblasts, increased amount of ADAM10 cleaves off high levels of sGPNMB, which acts through CD44, and subsequently downregulates *COL1A1* and *ACTA2* and exerts an anti-fibrotic effect. At the same time, sGPNMB forms a positive feedback loop to promote its own expression. CD44 appears to be modulated by histone acetylation in dcSSc fibroblasts.

In this study, we found a novel anti-fibrotic mediator in SSc. sGPNMB was capable of inhibiting the myofibroblast phenotype of dcSSc fibroblasts, including fibrotic markers, cell proliferation, and gel contraction properties. We also determined the molecular mechanisms involved in GPNMB expression and function in dermal fibroblasts (**Figure 5**). In normal fibroblasts, TGFβ downregulates GPNMB and CD44, and increases ADAM10, which is able to cleave off the soluble form of GPNMB from cell membranes. In dcSSc fibroblasts, increased amounts of ADAM10 cleave off high levels of sGPNMB, which then act *via* CD44, and subsequently downregulate COL1 and αSMA, and exert an anti-fibrotic effect. At the same time, sGPNMB forms a positive feedback loop in promoting its own expression. The futile anti-fibrotic effect of GPNMB in dcSSc fibroblasts, therefore, might be due to the suppressive effect of TGFβ on GPNMB and its receptor CD44. In addition, the low levels of sGPNMB released from these cells (pg/ml range) versus the dose needed (ng/ml range) for the anti-fibrotic effect might further reduce the anti-fibrotic effect of the sGPNMB-CD44 axis in SSc.

The role of GPNMB in tissue homeostasis and repair has been well documented. GPNMB is involved in ECM remodeling by inducing matrix metalloproteinases in fibroblasts (9, 16). Its ability to regulate immune responses and suppress fibrosis allowed it to promote regeneration in various injury models such as the muscle and liver (8, 17, 18). Interestingly the anti-fibrotic effect of GPNMB in part stems from its function in macrophages. It is suggested to promote an anti-inflammatory phenotype in macrophages by enhancing the M2 macrophages while inhibiting the M1 macrophages (19). Indeed, the involvement of GPNMB-mediated anti-inflammatory effect on macrophages was shown in kidney injury, adipose tissue, wound healing, and fibrosis (7, 19–23). In this study we revealed the novel anti-fibrotic properties of GPNMB, specifically its’ soluble form, in dcSSc skin fibroblasts. Considering the critical involvement of activated macrophages in SSc pathogenesis, the anti-fibrotic effect of GPNMB may be more pronounced in this disease if both fibroblasts and macrophages were included in the experimental system.

Our study also identified ADAM10 as the metalloproteinase responsible for the generation of sGPNMB by human dermal fibroblasts. GPNMB cleavage by ADAM10 was previously demonstrated in breast cancer cells (3). In our study, we found that ADAM10 was significantly upregulated in dcSSc fibroblasts, which is potentially mediated by TGFβ. Our results generated in skin fibroblasts are consistent with what was shown in lung fibroblasts by Lagares et al, where TGFβ induced ADAM10 expression (24). Interestingly, ADAM10-mediated proteolytic shedding of ephrin-B2 promoted fibroblast recruitment and activation as well as lung fibrosis (24). In addition, pharmacological inhibition of ADAM10 by GI254023X prevented bleomycin-induced lung fibrosis in mice. Although the effect of ADAM10 in SSc fibrosis has not been studied, the apparent pro-fibrotic effect of ADAM10 in other fibrotic conditions might imply that the anti-fibrotic effect of the ADAM10-mediated GPNMB shedding pathway plays a minor role in the overall pro-fibrotic phenotype observed in SSc due to the suppressive effect of TGFβ upon the sGPNMB-mediated anti-fibrotic effect.

We also identified CD44 as the receptor through which the ADAM10-sGPNMB pathway directs its anti-fibrotic properties in SSc fibroblasts. CD44 was first implicated as the receptor for sGPNMB by Yu et al. (25). They showed that sGPNMB released by macrophages stimulated ERK and AKT signaling in mesenchymal stem cells *via* engaging with CD44. Similar interaction between sGPNMB and CD44 was also shown in osteoclasts, astrocytes, and adipocytes (6, 26, 27). We now confirm that CD44 is the receptor for sGPNMB in skin fibroblasts. Interestingly, the dose-response curve of sGPNMB on gene expression in dcSSc fibroblasts appears to be a U-shaped curve. This typically occurs in receptor-ligand kinetics. At lower doses sGPNMB is effective, while at higher doses sGPNMB loses its stimulatory effect, possibly due to saturation of available CD44 on the cell surface. This phenomenon was similar to the effect of sGPNMB observed in keratinocytes (28). The dynamics of different functional endpoints appear to be different for the effect of sGPNMB. The maximal effective dose for sGPNMB-mediated gene expression changes appeared to be around 1 ng/ml. However, for proliferation and gel contraction assays, the maximum effective dose was around 100 ng/ml. At these higher doses, sGPNMB might require solicitation of other CD44-partnering proteins to mediate its effects.

By engagement with various ligands or colocalizing with many partnering proteins, CD44 has been shown to be involved in fibrogenesis and matrix remodeling (29–31). CD44 is critical for activation of TGFβ in a MMP-dependent manner, and also triggers fibroblast migration (32). CD44-null fibroblasts from acute lung injury exhibited decreased invasive and adhesive properties (33). In animal models of renal fibrosis, CD44 knockout mice had lower collagen levels, myofibroblast transformation, and TGFβ signaling (34). All of this points to a pro-fibrotic role of CD44. However conflicting results were observed in atherosclerotic and lung fibrosis models (35, 36). These results imply that the role of CD44 in fibrosis might be tissue and injury dependent. Interestingly, we showed that CD44 knockdown in dcSSc fibroblasts produced significantly lower levels of *COL1A1*. This might indicate that CD44, perhaps by engaging or partnering with other proteins, is pro-fibrotic in SSc. The role of CD44 in SSc fibrosis requires more in-depth examination.

One of the limitations of the study is that only *in vitro* findings were shown. Specifically, it is not clear whether sGPNMB levels in patient skin biopsies were altered. Although serum sGPNMB levels were similar between healthy controls and SSc patients, we showed that SSc fibroblasts secrete significantly higher levels of sGPNMB compared to normal fibroblasts. This implies that the amount of sGPNMB in the microenvironment in the skin is more critical than the levels in the circulation. In addition, we only focused on fibroblasts as a source for sGPNMB. It is unclear whether other cell types in the skin could shed sGPNMB. The relative contribution of each cell type to sGPNMB production, as well as the interaction of these cells in the context of the action of sGPNMB would be of interest in future studies.

## Conclusion

We identified a new pathway that inhibits myofibroblast activation in SSc, which involves cleavage of GPNMB by ADAM10 to generate soluble GPNMB that signals through CD44. Our data suggest that the anti-fibrotic effect of sGPNMB is suppressed by TGFβ in SSc. Enhancing sGPNMB production might provide a novel anti-fibrotic and therapeutic approach in SSc.

## Data Availability Statement

The original contributions presented in the study are included in the article/supplementary material. Further inquiries can be directed to the corresponding author.

## Ethics Statement 

This study was approved by the institutional review board of the University of Michigan, and each study participant signed an approved written consent prior to participating in this study. The patients/participants provided their written informed consent to participate in this study.

## Author Contributions

PP, LV, MG-R, EM, MO’m, SO, and SV: Performed experiments, edited manuscript for important intellectual content, and approved final manuscript draft. DK: Provided critical material, provided clinical evaluation and data, edited manuscript for important intellectual content, and approved final manuscript draft. P-ST: Study and experimental design, performed experiments, analyzed and interpreted data, wrote the manuscript. AS: Conceptualized the study, study design, interpreted data, wrote and edited the manuscript. All authors contributed to the article and approved the submitted version.

## Funding

This work was supported by the funds from the Donna and Larry Shelley’s Research Fund and the Edward T. and Ellen K. Dryer Early Career Professorship (Tsou), National Institute of Arthritis and Musculoskeletal and Skin Diseases grant K24 AR063120 (Khanna) and R01 AI097134 and R01 AR070148 (Sawalha).

## Conflict of Interest

The authors declare that the research was conducted in the absence of any commercial or financial relationships that could be construed as a potential conflict of interest.

## Publisher’s Note

All claims expressed in this article are solely those of the authors and do not necessarily represent those of their affiliated organizations, or those of the publisher, the editors and the reviewers. Any product that may be evaluated in this article, or claim that may be made by its manufacturer, is not guaranteed or endorsed by the publisher.
